# Motivation for Change and Alcoholism Treatment

**Published:** 1999

**Authors:** Carlo C. DiClemente, Lori E. Bellino, Tara M. Neavins

**Affiliations:** Carlo C. DiClemente, Ph.D., is a professor and chair of the Department of Psychology and Lori E. Bellino, M.Ed., and Tara M. Neavins, M.S., are graduate students in the Human Services Psychology Program and research assistants to Dr. DiClemente at the University of Maryland, Baltimore County, Baltimore, Maryland

**Keywords:** motivation, addiction care, stages of change, AODD (alcohol and other drug dependence) recovery, treatment, AOD (alcohol and other drug) use behavior, behavioral change, AODU (alcohol and other drug use) treatment method, motivational interviewing, intervention, patient treatment matching, literature review

## Abstract

Motivation plays an important role in alcoholism treatment by influencing patients to seek, complete, and comply with treatment as well as make successful long-term changes in their drinking. Both alcohol-abusing and alcohol-dependent people can be classified into different “stages of change” in terms of their readiness to alter their drinking behavior. Consequently, researchers have had to consider more seriously the role of motivation in the treatment of and recovery from substance abuse and to incorporate motivational enhancement strategies into treatment programs.

Motivation is an important first step toward any action or change in behavior. Sayings such as “You can lead a horse to water, but you can’t make it drink” reflect the fact that people generally will not perform desired behaviors unless or until they are motivated to do so. Until recently, many alcoholism treatment professionals used this approach when treating alcoholic patients, contending that interventions were useless until the alcohol-dependent patient was self-motivated to change his or her drinking behavior.

During the past several years, however, researchers and clinicians have shown increased interest in the concept of motivation and the role that motivation plays in recovery from alcohol problems. Researchers have outlined a series of stages of change to describe the process that a person goes through when making a behavioral change. Those stages—*precontemplation* (i.e., not yet considering change), *contemplation* (i.e., considering change but not taking action), *preparation* (i.e., planning to change), *action* (i.e., making changes in one’s behavior), and *maintenance* (i.e., changing one’s lifestyle to maintain new behavior)—offer a new perspective on motivation and the process of behavior change (DiClemente and Prochaska 1998; Prochaska et al. 1992). Recognizing that patients vary in their motivation or readiness to change, researchers have designed interventions and treatments to enhance motivation (DiClemente et al. 1992; Higgins and Budney 1993; Miller and Rollnick 1991; Miller et al. 1992; Stitzer et al. 1993). This article examines the concept of motivation and its influence on behavior change, the role of motivation in alcoholism treatment, and treatment methods designed specifically to influence motivation.

## Stages of Change and the Transtheoretical Model of Change

A number of studies (e.g., Carney and Kivlahan 1995; DiClemente and Hughes 1990) have demonstrated that people with alcohol and other drug problems who seek or participate in treatment differ significantly in their levels of motivation to change. Using an alcohol version of the University of Rhode Island Change Assessment (URICA) scale, DiClemente and Hughes (1990) reported on the various stages of change among alcohol-dependent patients seeking outpatient treatment. Patients in the precontemplation stage were more likely to deny that they had a drinking problem, stating, for example, “I am not the person with the problem. It does not make much sense for me to be here” or “As far as I am concerned, I do not have any [alcohol] problems that need changing.” Conversely, patients in the preparation and action stages were more likely to admit that they had a drinking problem, stating, for example, “I am actively working on my [alcohol] problem” and “I have a problem and I really think I should work on it.”

Using the same scale, Carney and Kivlahan (1995) found similar profiles among a large group of substance-abusing veterans. The same variations in motivation have been found in other treatment-seeking populations, including inpatient substance abusers (Isenhart 1994) and polydrug users in methadone maintenance treatment (Belding et al. 1995). Recognizing these differences was the first step to evaluating how differences in motivation affect participation in treatment programs and drinking outcomes.

Assessment of motivation presents a significant challenge. External influences and pressures, as well as internal thoughts and feelings, contribute to a person’s motivation both to consider and implment a change in behavior (Cunningham et al. 1994). Evaluating a person’s motivation requires assessment of the person’s attitudes and intentions, confidence and commitment, and decisionmaking ability (DiClemente and Prochaska 1998). Researchers have attempted to measure motivation in several different ways, including querying patients about their intentions and plans to change and asking multiple questions reflecting the different stages of change (DiClemente and Prochaska 1998; McConnaughy et al. 1989; Miller and Tonigan 1996; Rollnick et al. 1992). Other researchers have attempted to develop measures of motivation for treatment (DeLeon et al. 1997; Simpson and Joe 1993).

As an outcome of the revised perspective on the concept of motivation, clinicians and researchers are attempting to intervene earlier with problem drinkers and design programs to recruit and motivate unmotivated patients. Such programs are designed to address specific tasks and obstacles that arise at the different stages of change. To move from the precontemplation stage, the patient must admit to having an alcohol problem and recognize the need to change his or her drinking behavior. In the contemplation stage, the patient decides to change his or her behavior after weighing the positive and negative aspects of change. In the preparation stage, the patient increases his or her commitment to change and plans to take action. In the action stage, the patient develops specific behavioral strategies to change his or her drinking behavior. Finally, in the maintenance stage, the patient strives to avoid relapse by developing a lifestyle that supports the changes in his or her drinking. For successful recovery, patient motivation is important throughout the entire process, although it is an especially important focus during the first three stages.

## Motivation to Change and Motivation for Treatment

Motivation appears to be a critical dimension in influencing patients to seek, comply with, and complete treatment as well as to make successful long-term changes in their drinking (DiClemente and Scott 1997). Studies among substance-abusing patients have demonstrated the importance of motivation for treatment in predicting treatment participation and recovery (DeLeon et al. 1997; Simpson and Joe 1993). Motivation for changing problem behaviors like drinking, however, is not synonymous with motivation for participating in treatment. Many patients enter treatment under pressure from other people. Although these patients may attend treatment, they may not be ready to change their drinking behavior and may not actively participate in treatment.

Most substance abuse treatment programs and self-help initiatives are designed to assist patients who are ready to take action and address their problems. Depending on the type of program and the intensity of the examination procedures before admission, however, people who are not ready to change or who are in the early stages of change are often admitted into these programs. Therefore, most clinicians need to know how to handle unmotivated or reluctant patients who enter treatment and who are, at best, ambivalent about changing their behavior.

As pointed out by Miller and Rollnick (1991), traditional approaches to treating unmotivated patients with alcohol problems often use aggressive and confrontational strategies in response to the patients’ denial. In one widely used approach, a team of family members, friends, and colleagues unite to confront the drinker and convince him or her that alcoholism treatment is necessary (Johnson 1986; Liepman 1993). Recent evidence indicates, however, that confrontation can foster denial and resistance in the drinker (Miller et al. 1993). As Miller (1985) emphasized in his review of the motivation literature, clinicians who work with unmotivated patients must implement less confrontational and more motivation-generating treatment approaches.

## Sources of Motivation

Research investigating sources of motivation for change typically has compared intrinsic sources of motivation (e.g., feeling a sense of accomplishment) with extrinsic sources (e.g., financial incentives) (Deci and Ryan 1987). Generally, internal motivation is associated with greater long-term change than is external motivation (Deci and Ryan 1985). Curry and colleagues (1991) found that offering people financial incentives (i.e., extrinsic motivation) to stop smoking was less effective in both the short and long term than an intervention that enhanced smokers’ intrinsic motivation by encouraging and promoting personal responsibility.

Ryan and colleagues (1995) found that among people who received outpatient alcohol treatment, internal motivation (as assessed by a treatment motivation questionnaire) was related positively to both treatment involvement and retention. Among the study subjects, the outpatients with high levels of both internal and external motivation had the highest treatment retention and treatment attendance outcomes. Irrespective of their level of external motivation, outpatients with low internal motivation had the worst treatment outcomes. Finally, patients with more severe alcohol problems generally had greater internal motivation for treatment. The severity of the patient’s alcohol problems enhances internal motivation, presumably because the problem severity increases distress and thus influences decisionmaking.

Although internal motivation appears to be more effective for long-term success, external motivation seems to promote short-term abstinence from alcohol and other drugs. Interventions that offer financial incentives to patients who submit drug-free urine samples have been found to be significantly more effective than a standard treatment without financial incentives (Higgins and Budney 1993; Stitzer et al. 1993) (see the article in this issue by Higgins and Petry, pp. 122–127).

Relying solely on external pressure and incentives to influence a patient to modify his or her drinking behavior, however, can be difficult. A study of 263 inpatients in alcohol treatment found that patients whose motivations to enter treatment were related to current external threats (e.g., threatened loss of job, driver’s license, or spouse) experienced better treatment outcomes than did patients without such motivating factors (Krampen 1989). However, patients who had experienced one of the aforementioned losses in the past before entering treatment had poorer treatment outcomes than patients who had not experienced such losses. Because of the limited effectiveness of external motivators, treatment providers face the challenge of shifting patients’ motivation from external to internal incentives.

## Motivational Treatment Approaches

Treatment approaches designed to enhance patients’ intrinsic motivation include brief intervention, motivational interviewing, and motivational enhancement therapy (MET). Each approach is described in detail in the following sections.

### Brief Motivational Intervention

Brief (i.e., single-session) motivational intervention uses straightforward advice and information on the negative consequences of alcohol abuse to motivate patients to reduce or stop drinking. Although studies have demonstrated the effectiveness of minimal motivational interventions for alcohol-dependent patients in alcoholism treatment settings (Edwards et al. 1977) and non-alcohol-dependent patients in primary care settings (Fleming et al. 1997), this treatment approach has generally been viewed as more relevant for problem drinkers who are not yet alcohol dependent than for alcohol-dependent drinkers (Roche et al. 1995; Heather 1995).

Brief interventions vary in duration from one to four sessions, with each session lasting from 10 to 60 minutes. The goal of brief intervention is often reduced drinking rather than abstinence. Physicians or other treatment providers advise patients on the need to reduce their alcohol consumption and offer feedback on the effects of the patients’ drinking. The feedback is designed to increase patient motivation to reduce or stop drinking. For example, patients may be told that their current level of drinking puts them at risk for hypertension or liver dysfunction **(**U.S. Department of Health and Human Services [DHHS] 1997). Unlike more traditional treatment approaches, this technique does not involve overtly confrontational tactics but rather consists of a respected professional giving the patient advice and providing personally motivating information (Miller and Rollnick 1991). Refraining from explicit confrontation is thought to reduce patients’ defensiveness; because brief-intervention patients tend not to be self-referred and may not see any need for treatment, reducing this defensiveness is important (DHHS 1997).

Researchers generally have found brief intervention to be effective (DHHS 1997). For example, Bien and colleagues (1993) conducted a meta-analysis of 32 controlled studies of brief intervention for problem drinkers. Most of the patients in these studies were not alcohol dependent and were treated either in primary care or substance-abuse treatment settings. The researchers calculated and compared the studies’ effect sizes.^1^ When the researchers compared pretreatment and posttreatment drinking, they found that brief interventions effectively reduced drinking and yielded high average-effect sizes. In addition, when they compared brief-intervention patients with control group members, who were initially surveyed about their alcohol use but did not participate in a formal intervention, the strength of the effect of the intervention was reduced. This outcome indicates that merely asking people about their drinking and related behaviors may prompt some of them to reduce their drinking. A possible explanation is that increasing self-awareness of problematic drinking patterns by itself may be a motivating factor in changing drinking patterns. Finally, this meta-analysis showed that brief interventions were comparable in effectiveness to more extensive treatment.

More recently, Wilk and colleagues (1997) conducted a meta-analysis to explore the effectiveness of brief interventions with heavy drinkers. Examining brief interventions of less than 60 minutes, the researchers found that heavy drinkers who received brief interventions were nearly twice as likely to successfully reduce their alcohol consumption within the following year compared with heavy drinkers who did not receive brief interventions. This finding was independent of the client’s gender and the specifics of the clinical setting.

It is not clear whether all types of health care providers can be trained to offer brief motivational intervention. Successfully convincing providers to reliably offer this type of intervention to patients with alcohol problems is not always easy (Fleming et al. 1997). Specific training, however, does increase the frequency and effectiveness of brief motivational interventions. For example, Adams and colleagues (1998) found that specially trained providers who received less than 3 hours of formal brief-intervention training discussed alcohol use with their patients twice as frequently as health care professionals who had not received such training. Although these researchers did not directly test whether the brief interventions helped patients decrease their drinking, substantial support exists for the utility of brief interventions across primary care and clinical settings (Fleming et al. 1997; Wilk et al. 1997). (See the article in this issue by Fleming and Manwell, pp. 128–137.)

Further research is warranted to determine which patients benefit most from brief intervention (DHHS 1997). According to some evidence, heavy-drinking men may benefit more from brief intervention than from screening for alcohol problems (Anderson and Scott 1992; Babor and Grant 1992). For heavy-drinking women, however, brief intervention was not found to be superior to screening alone (Scott and Anderson 1991).

The patient’s level of motivation also may contribute to the effectiveness of brief interventions. For example, Spivak and colleagues (1994) found that among a group of highly motivated people who believed that they could reduce their alcohol consumption without treatment, three-fourths of them drank less after receiving a brief intervention in which they were only given a self-help manual with detailed instructions. In contrast, just over one-fourth of this group drank less after receiving materials consisting of general advice. Other researchers (Heather et al. 1993) have shown brief motivational interventions to be superior to skill-based approaches for patients with initially low motivation to change. Therefore, potentially beneficial future research may examine patients’ pretreatment level of motivation and other characteristics that may influence the effectiveness of brief intervention.

A third notable variable affecting the outcome evaluation of brief-intervention studies is the rate of attrition. Edwards and Rollnick (1997), for instance, reviewed all published studies of brief interventions conducted in primary care settings. The average attrition rate was 70.6 percent. Unfortunately, researchers rarely publish analyses that examine how participants who dropped out of the study or who were lost during followup differ from the participants who completed the study. Edwards and Rollnick (1997) found some evidence suggesting that compared with those who complete a study, lost participants tend to be younger (i.e., in their twenties and thirties), less educated, and heavier drinkers. Such patients may have less motivation, fewer resources, and additional complicating problems.

**Table:** Goals for MET Sessions

Session	Goal
Session 1 (week 1)	Provide personalized feedback from assessment instruments; identify and address ambivalence; build motivation for change
Session 2 (week 2 or 3)	Develop a change plan; strengthen commitment to change
Sessions 3 and 4 (weeks 6 and 12)	Review progress on the change plan; renew motivation; termination of therapy

Researchers are still investigating whether certain types of patients benefit more from brief intervention than do other patients. Such research could suggest whether these interventions can benefit patients with more severe problems or additional psychopathology. At present, brief interventions appear to work best to motivate middle-aged excessive drinkers with more serious, multiple problems to enter and participate in more extensive treatment.

### Motivational Interviewing

For less motivated patients with alcohol problems, a motivation-enhancing technique known as motivational interviewing (MI) may be more beneficial than either self-help books or cognitive-behavioral interventions (Heather et al. 1993). Based on motivational psychology and the stages-of-change model, MI focuses on enhancing and facilitating the patient’s internal motivation to change (Miller and Rollnick 1991). This approach assumes that the patient is responsible for changing his or her addictive behavior and recognizes ambivalence as a natural part of the process. In contrast to confrontational approaches, MI is designed to assist patients in working through their ambivalence and in moving toward positive behavioral change.

The MI therapist uses various techniques to help increase the patient’s motivation to change his or her behavior. One technique is reflective listening, a form of paraphrasing that enables patients to more fully tell their stories and to feel that they are being heard by the empathetic MI therapist. A second technique involves exploring the pros and cons of change, which may help patients realistically evaluate their behavior and current situation and, ideally, determine whether the pros of change outweigh the cons. A third MI technique, which supports the patient’s self-efficacy, or confidence that he or she can change, can help bridge the gap between a patient’s desire to change and concrete behavioral change. A fourth technique uses interview and assessment data to provide patients with personalized feedback regarding the problem behavior (e.g., comparing the patient’s level of alcohol use with national drinking norms) as a means of increasing self-awareness and of highlighting the discrepancy between the patient’s current behavior and the target behavior. Yet another technique involves eliciting self-motivational statements from the patient, such as recognition of the problem and concern for one’s own welfare. Self-motivational statements often propel patients to change as they reflect the topics of greatest concern to themselves. The MI therapist emphasizes the patient’s personal choice regarding change, deemphasizes diagnostic labels, and avoids arguing with and confronting the patient.

### Motivational Enhancement Therapy

The MET approach was specifically developed for Project MATCH, an 8-year, national, multisite, clinical trial initiated in 1989 that compared three alcoholism treatment methods and included a 39-month followup period (Miller et al. 1992).

MET combines MI techniques with the brevity of a less intensive intervention. MET consists of four treatment sessions over 12 weeks preceded by an extensive assessment. In the first session the therapist provides the patient with clear, structured, personalized feedback concerning his or her drinking frequency (number of drinking days per month), drinking intensity (number of drinks per drinking occasion), typical level of intoxication, risk for negative consequences of alcohol use, results of liver function and neurological tests, and risk factors for alcohol problems (e.g., familial risk and tolerance symptoms). This information comes from scores on various measures and diagnostic tests that the patient has completed before the session. The patient’s scores are then compared with the scores of a reference group of patients or other groups of American adults in order to increase the patient’s awareness of the extent to which alcohol has affected his or her life and to motivate the patient to change his or her drinking behavior. The patient receives a copy of the feedback report to take home.

During session 2, the therapist concentrates on strengthening the patient’s commitment to change by using MI techniques that are appropriate for the patient’s stage in the change process and on helping the patient develop a specific plan for change (e.g., what he or she will do, how he or she will do it, and who can help). During sessions 3 and 4, the therapist focuses on reviewing patient progress and renewing motivation and commitment by exploring remaining ambivalent feelings that the patient might have about changing the targeted behavior. Termination of the treatment and future plans are also discussed at the end of session 4, which involves a summary of the treatment progress. The therapist reviews motivational themes, summarizes the patient’s stage of change, elicits self-motivational statements for maintaining change, and explores future areas of change and resources for help. The goals for each session are summarized in the table on page 89.

## Project Match: Matching Alcoholism Treatments to Client Heterogeneity

Project MATCH consisted of two parallel but independent studies: one study was with patients who had received only outpatient treatment and the other study was with patients who had participated in either an inpatient or a day hospital treatment program and were currently receiving aftercare (Project MATCH Research Group 1997). The study was designed to test the effectiveness of matching patients to one of three conceptually different treatments based on various patient characteristics. Treatments included cognitive behavioral therapy (CBT), in which patients learned coping skills to reduce alcohol use; 12-step facilitation (TSF), which is based on the principles of Alcoholics Anonymous (AA); and MET. Each treatment produced significant and long-lasting reductions in alcohol consumption, and no single treatment was substantially more effective than another.

Project MATCH yielded several interesting results on the role of motivation in treatment. Motivation or readiness to change at the start of treatment (i.e., at baseline) was the most potent predictor of drinking outcomes throughout the posttreatment period for outpatients. During the final month of the 12-month followup period, less motivated outpatient clients in the MET group had a higher percentage of days in which they were abstinent from alcohol compared with less motivated clients in the CBT group. However, this effect was modest, consisting of a difference of 10 percent, or 3 drinking days per month, and was not evident at the followup conducted 3 years after treatment.

Although the study found little support for matching patients to treatments based on patient motivation, the results indicated that patients with different levels of anger had different treatment outcomes depending on the treatment they received. Outpatient clients who reported a higher baseline level of anger fared better after MET than after CBT and TSF treatments. Conversely, outpatient clients with low baseline levels of anger had better treatment outcomes after TSF and CBT compared with MET (Project MATCH Research Group 1998). No treatment matching effects with MET were found for aftercare clients (Project MATCH Research Group 1998).

DiClemente and colleagues (in press) investigated mediating factors hypothesized to account for the relationship between a patient’s initial readiness to change and his or her drinking outcome. They examined the client-therapist working alliance, treatment compliance, client processes of change, posttreatment readiness to change, and the client’s post-treatment self-efficacy with abstention. The researchers hypothesized that these variables influenced each other and formed a causal chain that would explain the link between motivation, treatment matching, and outcome. Although the study’s findings did not provide evidence of a causal chain for matching, the researchers found that patients who had greater motivation at baseline were more likely to have a strong client-therapist alliance and better posttreatment drinking outcomes across treatments. Baseline motivation levels were significant predictors of drinking outcomes for the entire year after treatment and at the 3-year followup for outpatient clients. Patients’ readiness to change at the start of treatment had a significant impact on their success in quitting and reducing drinking throughout the 3 years after treatment.

## Implications for Treatment Development and Evaluation

Motivation, a key element in treatment and recovery, influences a patient’s progression through the stages of change—from considering change, to making the decision to change, to following the planned action into sustained recovery. Current research and treatment initiatives reflect the increased focus on the role of motivation in alcoholism treatment. For example, most current clinical trials include measures of client motivation. The recent trials funded by the National Institute on Alcohol Abuse and Alcoholism (NIAAA)—Project MATCH and Project COMBINE, a study examining the combination of pharmacotherapy and psychosocial treatment—have included motivational measures and treatment components. Evaluations of substance abuse treatment programs commonly include motivational dimensions (Simpson and Joe 1993). The next few years should see dramatic growth in researchers’ understanding of the role of motivation, both intrinsic and extrinsic, in alcoholism treatment and recovery.

Efforts to intervene effectively with alcohol problems in primary care settings, court diversion programs, prison programs, and more traditional inpatient and outpatient treatment programs must address client motivation. In fact, as early identification and intervention programs become more proactive and aggressive, the importance of addressing patient motivation will only increase.

In general, motivated patients enter and attend treatment at higher rates than do less motivated patients. However, some extrinsically motivated patients may attend treatment regularly but be reluctant to participate in the treatment program. Other minimally motivated patients may attend and participate to some degree but fail to make substantial changes or sustain changes made in treatment. Both the type and intensity of the patient’s motivation for change are important potential moderators of treatment participation and recovery success.

An increasing number of treatment strategies and programs are being used to address the patients’ motivational needs. Some programs have established groups or initial program components to examine and increase motivation. Many substance abuse programs are incorporating MI techniques into their treatment repertoire, either by developing a separate motivational component or by incorporating those techniques into established treatments. Others are combining motivational interventions with cognitive-behavioral skills-based approaches and 12-step support group involvement.

Although research indicates that motivation-based approaches can increase patient motivation and improve drinking outcomes, researchers and clinicians still have much to learn about how to influence patient motivation, whether intrinsic or extrinsic. A number of issues would benefit from further research, such as the best way to measure motivation and whether primary care physicians can use motivational techniques to effectively treat patients with alcohol dependence as well as patients with less severe alcohol problems. A third important area of exploration is whether and how motivational techniques can be used with patients dually diagnosed with alcohol abuse or dependence and an additional psychiatric disorder. The next few years should yield interesting and important information about the feasibility and effectiveness of methods to motivate and move patients through the stages of change in all types of alcohol and other drug treatments, whether brief or more intensive.

## Footnotes

1 Effect sizes indicate the magnitude of the differences between the treatment group and the control group and show whether the treatment group has experienced a meaningful reduction in alcohol consumption or alcohol-related consequences compared with the control group.
